# Transesophageal echocardiography: a tool for intraoperative assessment of coronary blood flow

**DOI:** 10.1093/jscr/rjac603

**Published:** 2023-01-10

**Authors:** Pankaj Garg, Ishaq J Wadiwala, Lekhya Raavi, Nargis Mateen, Juan Crestanello, Si M Pham, Samuel Jacob

**Affiliations:** Department of Cardiothoracic Surgery, Mayo Clinic, Jacksonville, FL 32224, USA; Department of Cardiothoracic Surgery Research Unit, Mayo Clinic, Jacksonville, FL 32224, USA; Department of Cardiothoracic Surgery Research Unit, Mayo Clinic, Jacksonville, FL 32224, USA; Department of Cardiothoracic Surgery Research Unit, Mayo Clinic, Jacksonville, FL 32224, USA; Department of Cardiothoracic Surgery, Mayo Clinic, Rochester, MN 55902, USA; Department of Cardiothoracic Surgery, Mayo Clinic, Jacksonville, FL 32224, USA; Department of Cardiothoracic Surgery, Mayo Clinic, Jacksonville, FL 32224, USA

**Keywords:** transesophageal echocardiography (TEE), coronary artery obstruction, Bentall, aortic valve replacement

## Abstract

Transesophageal echocardiography (TEE) has become an indispensable part of cardiac surgery, but its potential for assessing coronary anatomy and blood flow remains underutilised. This case report presents a case of acute iatrogenic left main coronary artery obstruction following re-operative aortic valve replacement that was promptly diagnosed by intraoperative TEE and managed successfully by Bentall operation. We also emphasise the technique of TEE for coronary evaluation, its caveats and its clinical application during cardiac surgery.

## INTRODUCTION

Transesophageal echocardiography (TEE) is the point-of-care tool for the intraoperative cardiac evaluation [1, 2]. However, the potential of TEE for the assessment of coronary blood flow (CBF) is underutilised, and new onset global or regional wall motion abnormalities is used as a surrogate of myocardial ischemia [3]. Conventional coronary angiography remains the gold standard for the diagnosis of acute iatrogenic coronary artery obstruction (ICAO) [4].

We report a case of ICAO after third time re-operative aortic valve replacement (AVR). The diagnosis was confirmed on TEE by the presence of severely narrow left main coronary artery (LMCA) ostium with turbulent flow on color-Doppler and development of new-onset global left ventricular (LV) dysfunction. The patient was successfully managed by Bentall operation. We also briefly describe the technique of assessing CBF and its caveats.

## CASE REPORT

**Figure 1:**
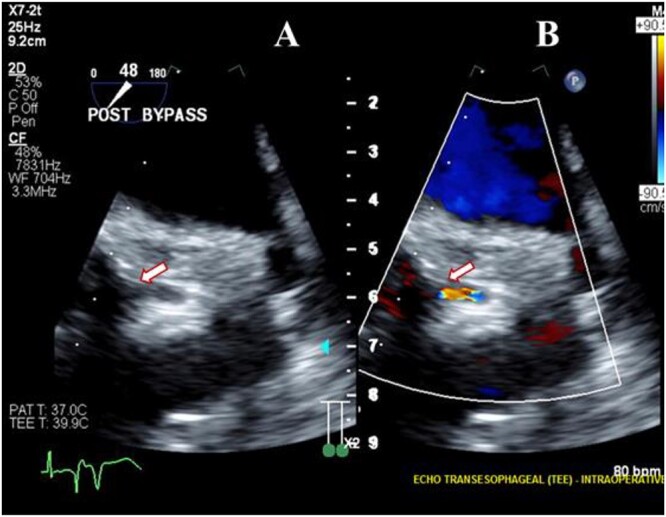
Transesophageal image following re-operative AVR showing severe stenosis (arrow) at the origin of the left main coronary artery (**A**) with turbulent flow (arrow) on color Doppler imaging (**B**).

A 56-year-old male underwent AVR for the third time with a 23-mm OnX valve under moderate hypothermic cardioplegic arrest for structural deterioration of bio-prosthetic aortic valve. After suturing the aortic prosthesis, the right coronary artery (RCA) ostium was about 1 cm above, whereas LMCA ostium was relatively close to the prosthesis. Following the removal of aortic cross-clamp, heart resumed activity in ventricular fibrillation and required multiple cardioversions before resuming the sinus rhythm. Subsequently, patient was weaned from CPB on high inotropic support. TEE revealed a well-functioning aortic prosthesis with no paravalvular leak. However, there was severe LV dysfunction with global hypokinesia. The right ventricular function was mildly reduced. A detailed evaluation of the coronary arteries demonstrated severe narrowing of LMCA ostium (Fig. 1A) with turbulent flow (Fig. 1B). A comparison with pre-bypass images confirmed this new finding, whereas RCA caliber and flow were comparable to pre-bypass images.

**Figure 2:**
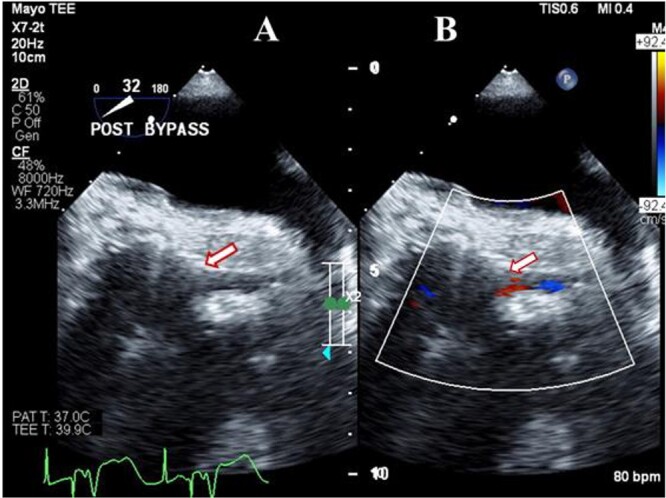
Transesophageal image following Bentall operation showing the widely patent (arrow) left main coronary artery (**A**) with Laminar flow (arrow) on color Doppler imaging (**B**).

As the flow in LMCA was suboptimal, options of coronary artery bypass grafting (CABG) the left anterior descending (LAD) and obtuse marginal arteries vs Bentall were considered. As harvesting the conduits for CABG would have further prolonged duration of LV ischemia; Bentall operation was performed with a 23-mm On-X valved conduit on moderate hypothermic cardioplegic arrest. Patient was weaned-off CPB on minimal inotropes. TEE confirmed the patent LMCA (Fig. 2A) with laminar flow on color Doppler imaging (Fig. 2B), normal ostium and flow pattern in the RCA and normal biventricular function. The patient made an uneventful recovery and was doing well at last follow-up 3 years after surgery. Last transthoracic echocardiography confirmed a well-functioning aortic prosthesis with good biventricular function.

## DISCUSSION

The aortic root and mitral valve surgeries are prone to ICAO [4]. Reported incidence of ICAO after AVR is 1–5% and increases with every re-operative surgery [5]. The severity of ICAO varies from mild stenosis to complete occlusion and commonly involves LMCA followed by RCA and rarely both [6]. Although the partial ICAO may remain completely asymptomatic, significant ICAO may result in severe ventricular dysfunction, intractable ventricular arrhythmias, inability to wean from CPB and circulatory collapse [5]. Therefore, a high index of suspicion, immediate diagnosis and appropriate management are crucial for a successful outcome. Further, transporting a hemodynamically unstable patient to catheterisation lab results in the delay in diagnosis and exposure to contrast, jeopardy of ischemic myocardium, and increased morbidity and mortality [7–9].

Advent of harmonic imaging, contrast agents and high-frequency transducers has made it possible to directly visualise the ostium and proximal part of all major coronary arteries on TEE to diagnose coronary artery anomalies, ICAO, measure CBF and CBF reserve [1, 10]. The role of TEE in the evaluation of ICAO has been reported in mitral valve surgery, transcatheter AVR and Bentall operation [11, 12]. Ours is the first report of TEE detection of acute iatrogenic LMCA stenosis and its successful management by Bentall operation. In our patient, TEE diagnosis of LMCA ostial stenosis with turbulent flow along with severe LV dysfunction not only expedited the management but also prevented prolonged myocardial ischemia and avoided a trip to the catheterisation lab.

Although the assessment of coronary arteries after AVR is challenging due to acoustic shadowing, LMCA and proximal 1 cm of the major coronary arteries can still be visualised in most patients. Ostium and the entire length of LMCA are visualised between 1 and 2 o’ clock positions in mid-esophageal aortic valve short-axis view (~45° omniplane) just superior to the left coronary cusp of the aortic valve. A slight change in the depth, probe rotation and omniplane allows following the course of LMCA to the bifurcation of LAD and the left circumflex (LCx) arteries. The RCA ostium can also be evaluated in the mid-esophageal aortic valve short-axis imaging plane between the 6 and 7 o’ clock position. With varying degrees of probe insertion, slight retroflection and rightward rotation, additional segments of the RCA become visible. Color Doppler flow imaging further improves the accuracy of detecting any stenosis or kinking of the coronary artery. Disordered blood flow due to ICAO frequently exhibits an aliased color Doppler pattern. Local color flow turbulence and aliasing seen in the coronary artery should be further interrogated with pulsed Doppler, simultaneously considering the cardiac cycle and the diastolic position of the coronary lumen. In the presence of ICAO, pulsed Doppler diastolic velocities are increased to 200–400 cm/s. Additional interrogation with continuous-wave Doppler should also be obtained for the maximal velocity. With combined 2D-echocardiography and color-flow pattern, TEE is quite sensitive in visualising flow in LMCA, proximal LAD (69–97%) and RCA (66–83%) [13].

Pulsed Doppler diastolic velocities in coronary artery >100 cm/s may not always signify ICAO as it may be elevated in patients with increased cardiac output, moderate-to-severe aortic regurgitation, aortic stenosis or hypertrophic cardiomyopathy. Therefore, simultaneous coronary artery lumen should always be evaluated with 2D-echocardiography.

In conclusion, TEE is an important point-of-care tool for the assessment of anatomy and CBF in major coronary arteries. New onset coronary artery stenosis with flow turbulence after cardiac surgery is an important indicator of significant ICAO. A routine evaluation of CBF in post-operative TEE can prevent catastrophic complications in at-risk patients, abate the trip to the catheterisation lab, expedite the patient management and improve the outcome.

## Data Availability

The data underlying this article will be shared on reasonable request to the corresponding author.
